# Evaluation and Comparison of the Geometrical Relationship of Tooth and Lip Arcs and Their Correlation to Smile Arcs Between Males and Females in a North Indian Population

**DOI:** 10.7759/cureus.34879

**Published:** 2023-02-11

**Authors:** Suchetana Basak, Arunoday Kumar, Gracy Panmei, Seltun S Anal, Rajesh S Nongthombam

**Affiliations:** 1 Prosthodontics, Crown and Bridge, Regional Institute of Medical Sciences (RIMS), Imphal, IND; 2 Prosthodontics, Crown and Bridge, Dental Private Practice, Imphal, IND; 3 Periodontics, Dental College, Jawaharlal Nehru Institute of Medical Sciences (JNIMS), Imphal, IND

**Keywords:** natural head position (nhp), lip arcs (la), tooth arcs (ta), esthetics, smile arcs (sa)

## Abstract

Background

With the increasing influence of social media, millennials and the generations that follow have increasingly pressing aesthetic concerns. Following this, there has been a sea change in treatment plans and procedures as well as the choice of material. Dentistry nowadays is dependent on digital data to compute and design prostheses; these technologies are often not readily available all over the world. The purpose of this research article is to measure the tooth arc (TA) and lip arc (LA) and their correlation to the smile arcs with a cohort consisting of males and females from a single center in northern India. The SA measurement, evaluation, and comparison of smile aesthetics in this young population may be used as a threshold to these smile variables so that prosthodontists can easily prevent or modify aesthetically displeasing features. This research article will be useful for cases requiring restorations and replacements of maxillary anterior teeth.

Materials and methods

In this research work, photographic analysis was used and photographs of hundred subjects were taken. The camera was fixed using a tripod, at an 11-inch distance from the face, so that a clear picture of the face could be taken from the tip of the nose to the chin. A digital single-lens reflex (DSLR) camera (Nikon D-60, with the Nikon DX AF-S Nikkor 18- 135mm lens, Tokyo, Japan) was used to take the facial photographs. As the posed smile is more predictable than the natural smile, subjects were asked to smile while keeping their natural head position (NHP). Parabolas were made with Math-GV software () and superimposed on the photographs to calculate the value.

Results

The mean LA shows greater curvature in the female population. There was no significant (p=0.92) relationship between TA curvature in the male (0.07±0.03) and female populations (0.08 ± 0.03), whereas the LA in males (0.10±0.03) and LA in females (0.12±0.04) showed a significant relationship (p=0.03) between both groups. The LA of the female population was found to be steeper than that of the male population. This study signifies that there is no significant difference (p=0.92) between the skeletal makeup of the male and female populations but the muscular activity and muscle function differ in the male and female populations. Regarding arc-wise comparisons in both male and female populations, the male population showed a statistically insignificant (p=0.27) correlation in lip and tooth arcs. But in the female population, the correlation between these arcs was statistically significant (p=0.01).

Conclusion

These study results provide useful guidance for evaluating anterior teeth and planning treatment for aesthetic restorative care. Clinicians should consider not only racial and gender differences when developing an aesthetic treatment plan but also the symmetry of the facial structure which should be in harmony with the dental arc. The harmony of each determinant of aesthetics, with each other, aids in promoting beauty as a whole.

## Introduction

Aesthetics comes from the Greek word ‘aisthetikos’, which means perceptive. Aesthetic appearance is always subjective and influences beauty and harmony ideals. Dental practice provides high-quality knowledge about different kinds of aesthetic dental restorations and cosmetics [1]. So, this field has an untapped potential to enhance the patient’s dental aesthetics using dental restorative materials, improved techniques, and elective cosmetic treatment [2]. For the application of cosmetic treatments, there is a need for deep knowledge of aesthetics. This knowledge can be easily used to predict the most efficient systematic technique to improve a particular deformation of aesthetics. Some of the aesthetic principles in smile designing are smile line placement (SIL) [3], minimized negative space (MNS) [4], axial inclination graduation (AIG) [5], incisal embrasure gradation (IEG) [6], and gingival frame contour (GFC) [7]. One scientific study of psychological research says that being perceived as beautiful makes individuals happier, more confident, and more successful than individuals who are considered to be less attractive. Modern research regarding improving the aesthetics of smiles has abounded. Goldstein reported that of 60 female beauty contestants, 97% perceived that they needed some aesthetic improvement in their dentition [8]. It is seen that patients are often motivated to seek dental care like restoring, enhancing, or creating aesthetics in today’s health and aesthetic-conscious world [9]. There are multiple factors that determine the attractiveness of a smile, and to restore it dentists must be cognizant of these factors [10].

The scheme of harmony and balance is followed to achieve the ultimate goal of a pleasing smile or to aid in recovering the compromised situations of a pleasing composition of a smile. The important parameters for a beautiful smile are facial aesthetics and gingival aesthetics as well as micro-aesthetic and macro-aesthetic principles [11]. The macro-aesthetic principles are symmetry across the midline, central dominance, and regressive proportions. It is known that beauty is subjective, so proportion was established for comparison. The value of proportion in aesthetics has been pointed out by many researchers like Aristotle, Pythagoras (who described the golden proportion), and the Greek geometrician Euclid [12]. In ancient Egypt, the golden number f (1.618) was developed, where the width-to-length ratio in the Egyptian rectangle is 0.6 (fV) [3]. The proportions are established and set out according to the Golden Number, sectio aurea, and Divine Proportion (phi=1,618.1). Golden proportion in dentistry was first documented by Lombardi which had a great role in developing aesthetics [13]. In addition, he defined the idea of a repeated ratio, which implies that in an optimized dentofacial composition from the frontal aspect, the lateral to central width and the canine to lateral width are repeated in proportion.

The two consecutive Fibonacci numbers tend to form the golden ratio as the series progresses to infinity. The Fibonacci series is 1, 1, 2, 3, 5, 8, 13, 21, and so on [14]. Each term in this series is the addition of the two previous terms whose proportion is the difference between the consecutive terms [15]. Frush and Fisher have given attention to the importance of the smile line being in the harmony with the curvatures of the incisal plane of the tooth arc (TA) and the superior border of the lip arc (LA) [16]. Also, Parekh et al. evaluated the computerized variations of smile arcs with a web-based study and reported that smiles with ideal and excessive smile arcs were significantly more acceptable than those with flat smile arcs [17]. Goldstein stated that in the determination of the arc, the midline is an essential factor [18]. Moskowitz and Nayyar mentioned that to maintain a perfect balance in aesthetics, analysis of the harmonious relationship in facial components in the smile should be in conjunction with the facial profile [8].

There are no evidence or analytical studies confirming which practice should be adopted for a particular facial form. So computerized analyses proposed by Goldstein affirms its importance, thus providing then dental practitioners to fulfill the patient’s expectations [2]. In addition to this, smile analysis has to be done with the determinants like dental midline, facial midline lip line, smile line, lines joining the corners of the mouth, and the golden proportion [4]. The standard of normalcy in an aesthetic smile is related to types of smiles like the papillary smile, gummy smile, or tooth to tooth smile, and the number of teeth displayed while smiling [3]. It is also related to the smile line wherein the curvature of the incisal edges of the maxillary anterior teeth coincides with the vermillion border of the lower lip or in a position just touching it while smiling [3]. The standard may serve as a guideline for the restoration or enhancement of aesthetics for the anterior component of the dentition [3]. The purpose of this research article is to measure and compare the TA and LA between young north Indian male and female populations to achieve the restoration of pleasing smiles, which is one of the various parameters of smiles.

## Materials and methods

**Figure 1:**
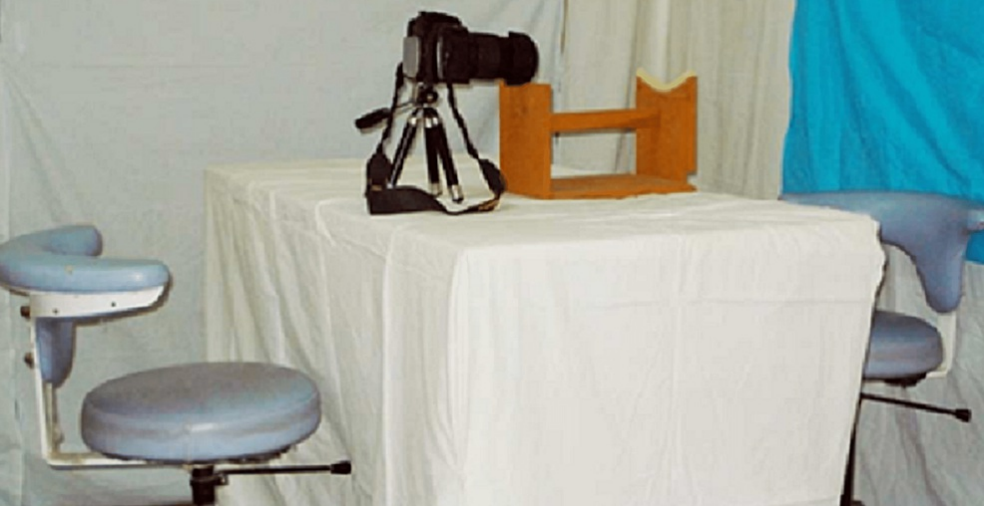
Photography Set up

**Figure 2:**
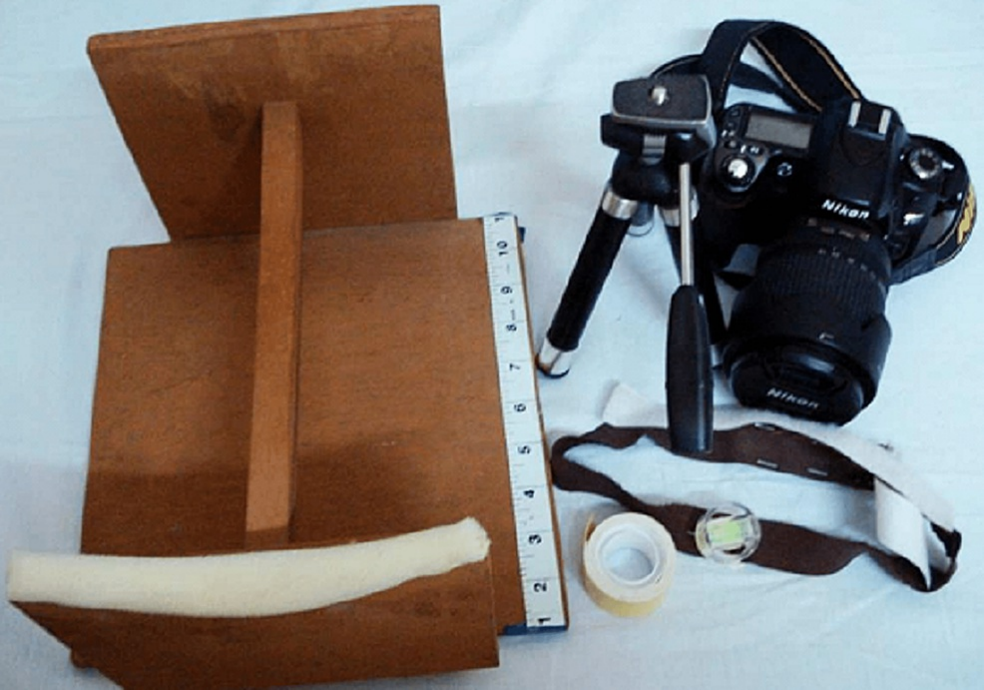
Armamentarium for photography

In this study, photographs of 100 subjects (50 male and 50 female) were taken and analyzed. The sample size was decided based on the literature surveys and also based on the type of statics analysis used and comprised students of Rajasthan Dental College in the age group of 18-30 years. Subjects were included who satisfied the inclusion criteria. This research work was carried out to evaluate, measure and compare the TA and SA of the male and female populations in the North India region from frontal view photographs. As the study involved human subjects, ethical clearance was provided by Rajasthan Dental College and Hospital, Rajasthan, India which is affiliated with Rajasthan University of Health Sciences, Jaipur, India with the ethical clearance certificate number RDCH/Ethical/2021/011. The photographs of the smile of the patients were taken using the Digital Single Lens Reflex (DSLR) camera (Nikon D-60- with lens Nikon DX AF-S Nikkor 18-135mm, Tokyo, Japan). Figure 1 shows the photography setup and Figure 2 presents the armamentarium.

**Figure 3:**
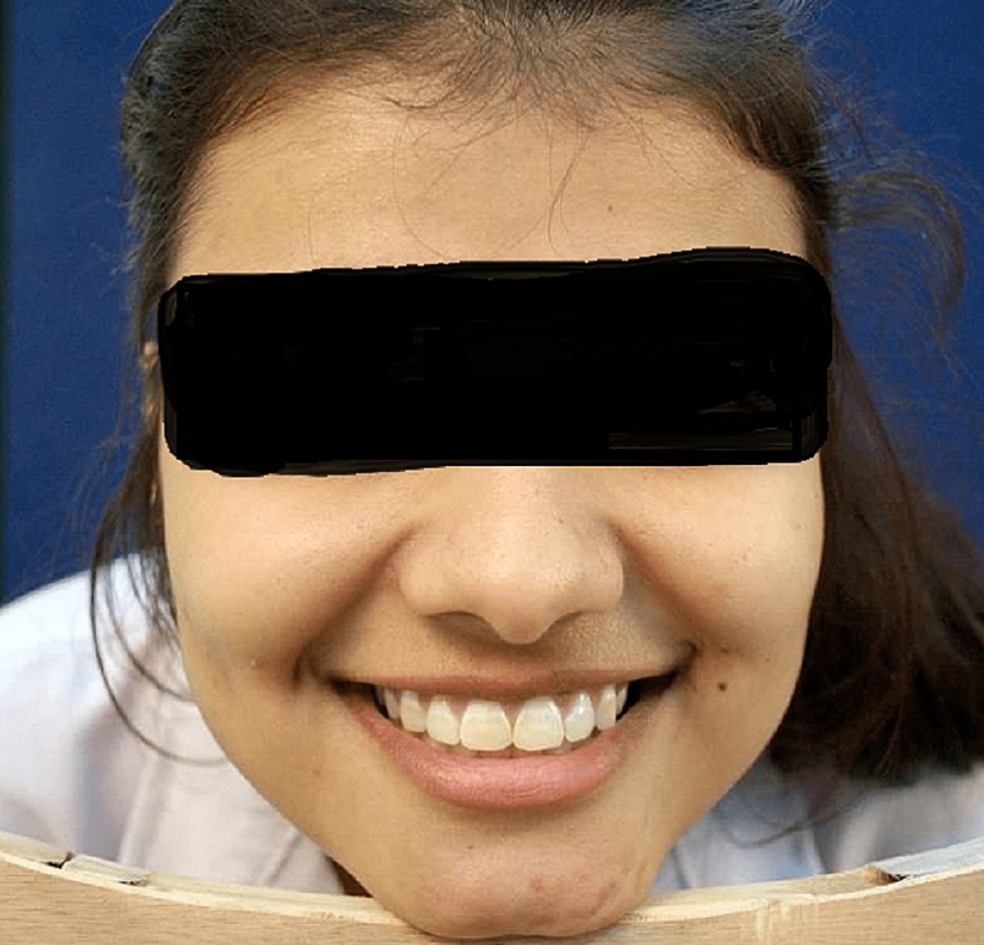
Smile view as seen through the viewfinder of the camera

 The used camera accessories are a tripod stand, blue background, and custom-fabricated stand (for a fixed subject-lens distance). Figure 3 shows the smile view as seen through the viewfinder of the camera.

**Figure 4:**
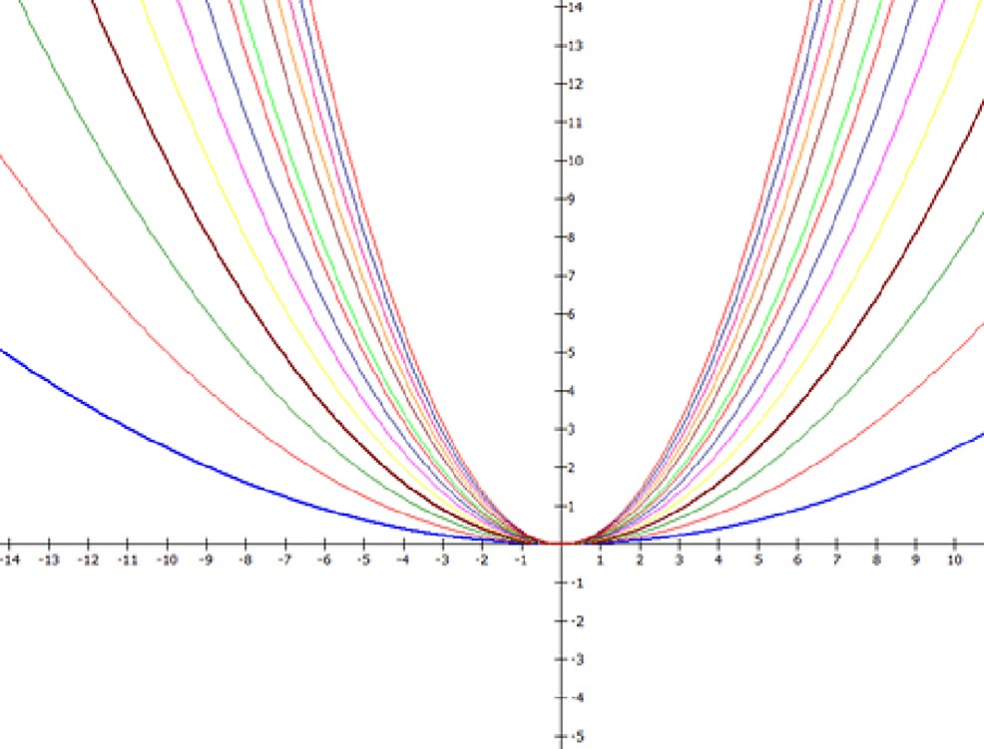
Smile view by graphing of the photograph using MathGV software MathGV software:

Figure 4 shows the graphing of the photographs using the software MathGV4 v. 4 () and Adobe Photoshop 7 Professional software (Adobe Inc., San Jose, CA). The fluid level device or spirit level device with t-sided adhesive tape and Velcro band is used. The diagnostic instruments used are the mouth mirrors, and the probes [19]. 

In this work, 100 subjects (50 male and 50 female) were selected between the age of 18-30 years from north India, and their photographs are taken for evaluation of images using computer software. Then, the comparative evaluation of TA and LA was done. The subjects were considered based on some criteria; for instance, individuals with apparent aesthetic/ pleasing smiles with no overlapping, no spacing, no crowding in the anterior teeth, overbite, and overjet were considered aesthetically acceptable, with no remarkable malformations, discolorations, and structural deformities. Unpleasant color or contour of gingiva was not preferred. The subjects with any missing permanent teeth other than third molars, any retained deciduous tooth, history of orthodontic treatment, and any tooth size alteration or anterior restorations and/or plastic or maxillofacial surgery were not considered.

All the subjects were informed about the nature of the study and their consent was taken. The subjects to be photographed were made to achieve the natural head position by looking at a distant point. Natural head position (NHP) represents a valid craniofacial reference system with larger interindividual and intraindividual reproducibility compared to other planes. It was marked using a spirit level device (fluid level device) on the head using a double-sided adhesive and a Velcro band. The repeatable and reliable head position (19, 34 NHP) is used in the study [20]. Once the desired position was achieved and confirmed, the subject was made to place his/her chin on the custom-made stand, maintaining the same head position, and the photograph was taken, using the grid system of the camera.

**Figure 5:**
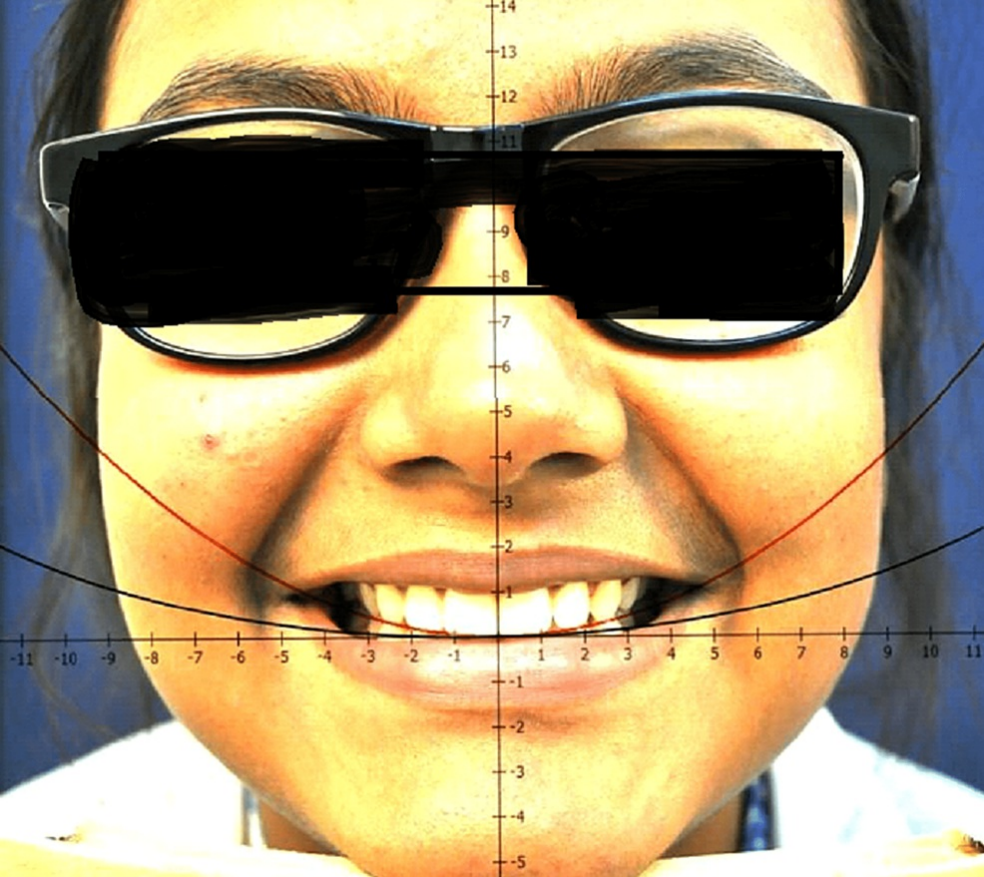
Evaluated photograph with Abode Photoshop Professional 7

A smile view was taken at a fixed focal length of 100 mm at a fixed subject-lens distance of 11 inches using a custom-made wooden stand. The stand was made such that the distance between the subject and lens remained constant; the subject rests his/her chin over the chin support of the stand, and the camera was placed on the tripod on the other end of it. The grid system of the camera was used to position the lens with a central focus point focusing at the incisal embrasure between central incisors. A face view was clicked to confirm the head position of the subject against a blue background. All the photographs were then analyzed using MathGV4 and Photoshop. Figure 5 shows the photographs evaluated with Photoshop. Curves were generated with MathGV software as parabolas of the formula f(x)= nx2 where, the value of "n" is taken from 0.025 to 0.350, with an increase in the value of 0.025 in progression [15]. An increased value of n in the equation means a steeper curve.

The curves generated from Math-GV4 software were overlayed on the participant's photograph using Adobe Photoshop and the best match for the tooth and lip arch was evaluated based on the observers' perception. The means of TA and LA for both genders were calculated. Differences between the means were tested. Statistical analysis was done for these values to compute the mean value of TA and LA for both genders. The effect of gender on the TA and LA curvature and the relationships between both of them were checked.

## Results

The smile view photographs of 100 subjects (50 males and 50 females) aged between 18-30 years were evaluated using Math-GV software and Abode Photoshop Professional 7. The TA and LA were measured by using the software and the data collected were subjected to statistical analysis. For statistical analysis, mean, standard deviation (SD), t-test, and paired t-test is performed and comparative evaluation is done between male and female. The mean is determined using the formula:

$$mean= \frac{\Sigma x} {n}$$

 

The apparent mean of TA and LA for males and females are calculated and given in Table 1.

 $SD=\sqrt{ \frac {\Sigma(x_{i}-x^{-})^2} {n-1}}$ 

Where, xi = individual observation in the groups, x− = mean, n = number of samples

**Table 1:** Mean Apparent Tooth Arc and Lip Arc in Males and Females

Smile Arc	Male	Female
Tooth Arc	0.07	0.08
Lip Arc	0.10	0.12

The mean ± SD of males for the range 0.04-0.15, 0.04-0.18, and of females for the range 0.03-0.17, 0.05-0.18 are recorded in Table 2.

**Table 2:** Tooth and lip arc measurements between male and female subjects

Arc	Male		Female	
	Range	Mean± Standard Deviation	Range	Mean± Standard Deviation
Tooth Arc	0.04-0.15	0.07±0.03	0.03-0.17	0.08±0.03
Lip Arc	0.04-0.18	0.10±0.03	0.05-0.18	0.12±0.04

The t-test is used to determine whether there was a statistical difference between males and females in the parameters measured. It is obtained by calculating as per the following equation:

$t= \frac {\bar x_{1}- \bar x_{2}} {s \sqrt{\frac {1} {n_1}+ \frac {1} {n_2}}}$ ​​​

$s^{2}= \frac {(n_{1}-1)s_{1}^{2}+ (n_{2}-1)s_{2}^{2}} {n_{1} +n_{2}-2}$ 

where, x¯_1 _− x¯_2_ is the mean of two samples, s is the pooled SD, n_1_ and n_2_ are the sample sizes, s_1_ is the variance of the first sample and s_2_ is the variance of the second sample, which is mathematically defined by equation.

 And the paired t-test is performed to determine whether there were differences between the right and left measurements on the parameter using the equation as follows:

 $t= \frac {\bar x} {s \sqrt \frac{1}{n} }$

For the arc-wise comparison between TA and LA in the male population, the t-value is 1.11, and for the female population, it is 2.77. For the gender-wise comparison (male vs female), in the case of TA, the t value is 0.11, and for LA it is 2.28 (Table 3).

**Table 3:** Comparison of various parameters (arc wise and gender wise)

Comparison of various parameters	Arc wise		Gender wise		
Arc	Male	Female	Male Vs Female		
	Mean ± Standard Deviation	Mean ± Standard Deviation	Mean Difference	t-test value	p-value (level of significance)
Tooth Arc	0.07±0.03	0.08±0.03	0.01	0.11	0.92 (not significant)
Lip Arc	0.10±0.03	0.12±0.04	0.02	2.28	0.03 (significant)
Mean Difference	0.03	0.04			
t-test value	1.11	2.77			
p-value (level of significance)	0.27 (Not significant)	0.01 (significant)			

$\bar x$ is the mean difference in each set of paired observations, s is the SD of the difference and n is the number of observations. In the entire above test, the “p” value of less than 0.05 is taken as the statistical significance. Data analysis is carried out using the Statistical Package for Social Science (SPSS, V 10.5) package and the comparison of different parameters of male and female are tabulated in Table 3.

The correlation analysis with Pearson's Correlation Coefficient (r-value) and t-test (p-value) is compared and given in Table 4.

**Table 4:** Correlation coefficient analysis between TA and LA in male and female

Male		Female	
r value (Pearsons Correlation Coefficient)	p value (level of significance)	r value (Pearsons Correlation Coefficient)	p value (level of significance)
0.66	<0.001, Highly Significant	0.59	<0.001, Highly Significant

## Discussion

This study aimed to investigate the existence of the geometrical relationship between TA and LA and evaluate their correlation with respect to the aesthetics of a north Indian population. As we know, an attractive smile line is one of the most essential features of a pleasing smile. In order to have “pretty smiles”, various factors like teeth morphology and color, gingival and lip contour, texture and color, facial form and color, etc. and their relationship to each other has to be taken into consideration for smile designing [11].

Evaluating and analyzing the photograph of an individual can be of great help to determine how the lip and teeth can make up a good smile during talking, smiling, or laughing [21]. Photographic analysis can determine how the lips and soft tissue frame the smile in different positions of speech, smiling, and laughing [19]. Therefore, in this research work, photographic analysis is used and photographs of 100 subjects were taken.

The camera was fixed using a tripod at an 11-inch distance from the subject so that a clear picture of the face could be taken from the tip of the nose to the chin. A DSLR camera was used to take the facial photographs. NHP has been proposed as a preferred reference position for assessing facial morphology. After the patient's head is fixed at NHP, they are asked to smile while looking at a fixed point as the photograph is shot [21].

Several authors, using different methods, have conducted NHP studies and their data agree on the consistency of an individual’s head posture over time [19]. In order to have an aesthetically pleasing restorative smile, the geometrical relationship between the LA and TA has to be determined [21]. It would also be helpful if statistically reliable relationships existed to support the existing relationship theories [22-25] as facial measurements in terms of aesthetics can be difficult and misleading due to the variability of the intra-cranial reference lines according to the diversity of head postures. In order to have an aesthetically pleasing smile, the morphology and its location in the TA which is specific for each and every tooth and the gingival contour, texture, and color have to be considered when designing smiles [3]. The inter-relationship between the teeth, the gingivae, the lip, and the patient’s facial form and color creates a dynamic macro-aesthetics of an individual [2].

A dentist in collaboration with the dental technician works together hand in hand to give an aesthetic and pleasing dental smile to an individual who requires prosthetic rehabilitation. Macro-aesthetics helps to co-relate the relationship between the maxillary anterior teeth and its neighboring soft tissues like gingivae and lip areas so as to have an aesthetic and pleasing facial and dental smile. In this study, we have applied the theories of macro-aesthetics to a greater extent. 

A paper by Ramirez discusses the research of Fayyad, Ward, Mashid et al., Preston, Hasanreisoglu, and others who have used photographs for the evaluation of teeth and facial dimensions [12]. These provide ample time and ability to measure dimensions and proportions and hence were chosen in this study as an aid in measurement. The idea that for the anterior teeth arrangement, maxillary incisal edges should coincide or follow or just touch the curvature of the lower lip while smiling is considered [3]. The patient is instructed to perform a static and perfectly reproducible smile. It has been recommended that smile photographs be standardized with a static smile, due to its reproducibility, in the NHP [21]. In order to have an aesthetically pleasing smile, the midline of the face should coincide with the dental midline.

An individual can have a papillary smile, gummy smile, or teeth-to-teeth smile depending on the lip length and mobility, crown length, or vertical maxillary skeletal length while talking, smiling, or laughing [3]. So, these factors are considered during smile photograph analysis. In the present study, the LA was found to be steeper than the TA in both genders. LA of males is 0.10±0.03 and LA of females is 0.12±0.04, whereas TA of males is 0.07±0.03 and TA of females is 0.08±0.03. The mean tooth arcs of females had greater curvature than males. In the case of LA, the means of lip curve for both genders were 0.10 for males and 0.12 for the female population. The mean of LA shows greater curvature in the female population. There was no significant (p=0.92) relationship between TA curvature in the male and female populations, whereas LA in the male and female populations showed a significant (p=0.03) relationship between both groups. The LA of the female population was found to be steeper than the LA of the male population. This study signifies that there is no significant difference (p=0.92) between the skeletal makeup of the north Indian male and female population but the muscular activity and muscle function differ in the male and female populations. In arch-wise comparison in both the male and female populations, the male population showed a statistically insignificant (p=0.27) relation in lip and tooth arc. But in the female population, the correlation between these arcs was statistically significant (p=0.01). Aesthetics is a phenomenon of the intellect, in its broad sense. It differs from person to person and community to community perception.

This study's results provide useful guidance for evaluating anterior teeth and planning treatment for aesthetic restorative care. Clinicians should consider racial and gender differences when developing an aesthetic treatment plan. One of the primary goals of prosthodontic treatment is to attain and preserve optimal facial attractiveness. This trend is reflected by the growth of aesthetic dental procedures, converting dentistry from a purely healthy profession to one of health and elective aesthetic services. With the variation that is present in nature, the final aesthetic outcome is rarely based upon or follows the mathematical principles of designing smiles. This approach serves as a platform on which to base the initial smile design procedure. However, on understanding the relationship between beauty, mathematics, and the surrounding world one begins to appreciate their interdependence.

The limitation of the study is the parabolic equation f(x)=nx^2^, the value of "n" varies from 0.025 to 0.35, with an increase in value of 0.025 in the progression considered in this study [15]. But for some patients having variations in LA and TA, where the value of n would be less than 0.5 and greater than 0.35, then for such cases, studies are required. Further, the curves generated for the MathGV4 software were overlaid on the participants' photographs and the best fit was evaluated based on the observer's perception, which would vary from person to person.

## Conclusions

One of the primary goals of prosthodontic treatment is to attain and preserve optimal facial attractiveness. There are several parameters in smile evaluation but harmony between the curvatures of the incisal edges of the maxillary anterior teeth (the tooth arc) and the superior border of the lower lip (the lip arc) is of utmost importance for esthetic rehabilitation.

This study concluded that there was no significant difference in skeletal parameters and teeth alignment in the North Indian male and female population who comprised our cohort. But when it comes to the muscular factors, the female population showed a statistically significant difference. The lip line of the females was significantly steeper than those of their male counterparts. Even in the same gender, the female population had steeper lip arc than tooth arc.

Aesthetics is a qualitative term, which varies in perception depending on ethnicity and geographical location. So, more studies should be conducted on different smile parameters and their correlation to aesthetics for the subjects of the different geographical areas of India and other countries.
